# Prevalence of essential tremor in urban Lagos, Nigeria: a door-to-door community-based study

**DOI:** 10.1186/1471-2377-12-110

**Published:** 2012-09-27

**Authors:** Njideka U Okubadejo, Idowu A Bankole, Oluwadamilola O Ojo, Frank I Ojini, Mustapha A Danesi

**Affiliations:** 1 Neurology Unit, College of Medicine, University of Lagos and Lagos University Teaching Hospital, Idi Araba, Lagos State, Nigeria; 2 Department of Medicine, Irrua Specialist Hospital, Irrua, Edo State, Nigeria

**Keywords:** Essential tremor, Prevalence, Nigeria, Subsaharan Africa

## Abstract

**Background:**

Essential tremor (ET) is one of the commonest movement disorders though the prevalence varies globally. There is paucity of data on ET prevalence in sub-Saharan Africa. The study aimed to determine the prevalence of ET in a Nigerian community.

**Methods:**

This door-to-door survey was conducted in two stages. In Stage 1, 3000 randomly selected residents of an urban centre in Lagos, Nigeria, were screened using a questionnaire to detect symptoms of movement disorder. 234 participants who responded positively regarding presence of tremors were rescreened using an ET-specific questionnaire, a face-to-face interview and neurological examination. Diagnosis of ET was based on the Movement Disorders Society (MDS) consensus diagnostic criteria for ET.

**Results:**

Of the 3000 participants, forty responded positively to the ET screening questionnaire, of which 36 (19 females and 17 males) had a final diagnosis of ET, giving a crude prevalence of 12 per 1000 (95% CI = 8.1- 15.9). Gender specific prevalence was 10.3 /1000 in males and 14.3/1000 in females. Age specific prevalence increased with advancing age in both sexes. Age adjusted prevalence (WHO New world population) was 23.8 per 1000.

**Conclusions:**

We documented a high prevalence of ET in this study, with typical increasing prevalence with advancing age as previously reported in other populations.

## Background

Essential tremor (ET) is the most common pathologic tremor in humans and one of the most frequently encountered movement disorders in clinical practice. The world-wide prevalence of ET ranges from 4.9 – 39.2 per 1000 across different populations, and is highest in persons above 60 years of age (13.0 – 50.5 per 1000) [1]. Traditionally viewed as a single disease entity, ET is now considered to be a family of distinct diseases based on the clinical phenotypic variability, including occurrence of other tremors, and additional motor and non-motor features (mainly cognitive, somatosensory and psychiatric) [2].

ET is characterized by an action (postural or kinetic) tremor that is clearly visible, continuous, bilateral and usually symmetrical. The tremor often involves the arms, head and/or voice, with occasional affectation of the legs, chin, and trunk [3]. The prevalence of ET increases with age and appears to be higher in Caucasians than African Americans even within the same geographic location [4]. ET occurs at all ages although there is a bimodal peak in the second and sixth decades [5]. Disease progression is slow, occurring over several years although the factors contributing to its progression are unknown.

Other motor features may be encountered, particularly with advancing disease, and include gait ataxia, postural instability*,* and eye movement abnormalities [6,7]. These features mimic cerebellar disorders and lend credence to the hypothesis that the pathogenesis of ET is related to cerebellar dysfunction. Non-motor accompaniments such as cognitive impairment particularly executive dysfunction [8], sensory abnormalities such as mild olfactory dysfunction [9], hearing impairment [10], mood disturbance – anxiety and depression [11], and social phobia [12] have also been reported in association with ET.

ET is associated with significant functional disability particularly in persons with an upper limb tremor [13]. 15 – 25% of ET patients seen clinically retire prematurely and 60% do not apply for work or promotion because of uncontrollable tremors [14]. Clinical heterogeneity in the severity of ET contributes to a diagnostic gap, with patients only tending to present in the event that disease progression interferes with activities of daily living or results in social or functional impairments. Thus, the prevalence of ET derived from hospital-based studies will reflect an under ascertainment. There are few community-based prevalence figures for ET in Africa. In a community based study of neurological disorders in Nigeria by Osuntokun *et al.* in 1982 [15], the crude prevalence of ET was reported as 10/100,000 while Haimanot *et al.* from Ethiopia reported a crude prevalence of 5/100,000 [16]. More recently, Dotchin *et al.* (2008) reported an ET prevalence of 82/100,000 from a community based study in Tanzania (east Africa) [17].

The present study was designed to determine ET prevalence in Nigerians from a community-based methodologically sound perspective, providing a credible basis for comparison with other populations.

## Methods

The study employed a multi-staged design incorporating a population-based door to door screening and a face-to-face interview. The population survey was conducted in Surulere Local Government Area (LGA), Lagos State, Nigeria between March and September 2008. The study was tagged onto a prospective community based study on the epidemiology of neurological disorders in Lagos State, Nigeria [18] by taking advantage of the research funding and infrastructure for the study. Surulere LGA is a mixed-income urban community in Lagos State, with an estimated population of 750,000 (based on data from the National Population Commission of Nigeria), comprised of approximately 750 enumeration areas (EAs), with each EA constituting of 1000 persons. 2% of the enumeration areas (estimated population of 15000) were previously randomly selected (based on a computerized random-selection program) and were delineated in the Epidemiology of Stroke in Lagos (EPISIL) study [18].

Ethical approval for this study was obtained from the Health Research Ethics Committee of the Lagos University teaching Hospital. Written informed consent was obtained from all study participants. The study team was comprised of 4 neurologists and 5 trained field assistants. The study was conducted in 2 stages.

### Stage 1 - Screening for movement disorders

In the 1^st^ stage of this study, 20% (3000 persons) of the original population used for the EPISIL study were randomly selected and rescreened for the presence of movement disorders using a movement disorder screening questionnaire developed for the purpose of the study. 3 screening questions were used to detect potential cases of movement disorders;

 a) Do your hands and/or arms shake?

 b) Have you noticed you have become slower when walking?

 c) Do you shuffle your feet (drag your feet on the floor) or take small steps when you walk?

Response options were “yes”, “no”, “don’t know” and “didn’t respond”. A total of 234 participants responded positively to the question regarding presence of tremors in the screening questionnaire and were screened in stage 2 of this study.

### Stage 2 - ET screening and case ascertainment

**Figure 1:**
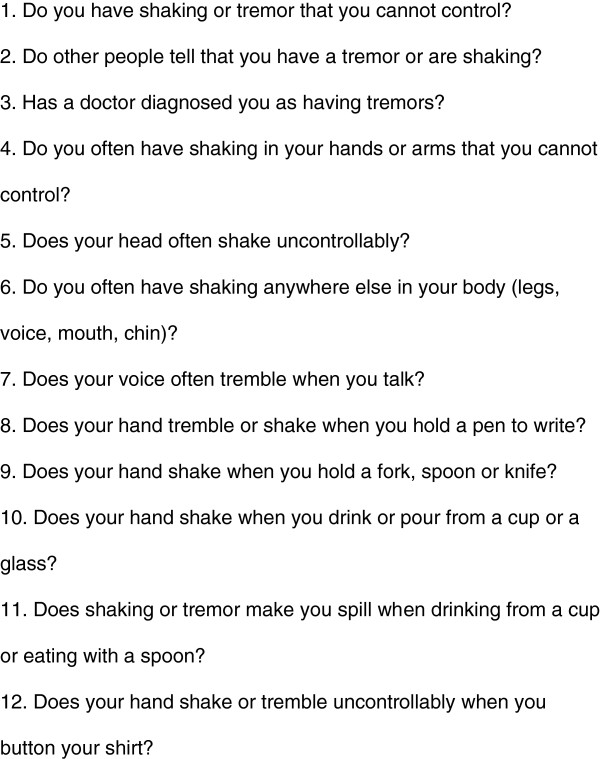
** List of questions in12-item ET specific questionnaireused in stage 2(case ascertainment).**

In the second stage, all 234 participants who screened positive for tremor in Stage 1 were rescreened by a neurologist in the participant’s home, using a 12-item ET specific questionnaire (Figure 1) and a questionnaire to document their demographic and historical data. In addition, a specific face-to-face interview was conducted to differentiate between ET, other tremors and parkinsonism. Specific aspects of this specific interview included questioning about difficulty rising from a low chair, change in handwriting, softening of the voice, poor balance, mouth hanging open and drooling of saliva, difficulty doing tasks like buttoning a shirt or using a screwdriver, walking with a stooped posture and feet being glued to the floor. All the participants in stage 2 also had a neurological examination conducted by a study neurologist to further ascertain the clinical diagnosis of ET. The diagnostic criteria for ET were based on the Movement Disorders Society (MDS) consensus diagnostic criteria [19]. ET was diagnosed in the presence of both major criteria (bilateral action tremor of the hands and forearms or isolated head tremor without dystonia) in the absence of any other neurological signs. Participants with parkinsonism, cerebellar (intention) tremor, drug-induced tremors, dystonia and tremors in the presence of features of hyperthyroidism were all excluded from the study. 

Severity of ET was assessed using an essential tremor scale in which tremor was rated as 0 (absent), 1 (slight, infrequent, mild tremor, writing and drawing of spiral minimally impaired), 2 (moderate – bothersome, writing and drawing of spiral moderately impaired), 3 (severe – writing and drawing severely impaired; interferes with many activities such as drinking liquids), or 4 – (markedly severe – interferes with most activities) [20].

### Performance of the ET instrument (sensitivity, specificity, and number needed to diagnose)

The sensitivity and specificity of the ET screening instrument was determined using the formula sensitivity = true positive/ true positive + false negative. The specificity was calculated as being equal to true negative/true negative + false positive.

Of the 40 who screened positive to the ET specific questionnaire in stage 2, 36 were finally diagnosed as ET (true positive), while 4 were not ET (false positive). These four were excluded based on the following: Parkinson’s disease – 1, drug induced parkinsonism – 1, drug induced tremor (salbutamol tablets for bronchial asthma) – 1, and hyperthyroidism – 1. The 80 non ET participants used to assess the performance of the ET specific instrument were randomly selected from the non ET following rescreening with the ET specific instrument and face to face examination. All did not have ET based on the diagnostic criteria (including hand tremor and head tremor), resulting in false negative of 0 and true negative of 80.

The total numbers in each category were thus: true positive – 36, true negative – 80, false positive – 4, and false negative – 0. Thus, the sensitivity of the ET screening questionnaire was 100%, and the specificity was 95.2%. In addition, the utility of the screening instrument was further evaluated using number needed to diagnose (NND) defined as the number of patients that need to be tested to give one correct positive test (Youden’s J = 1/[sensitivity – (1-specificity)] [21]. The NND ET using the ET specific questionnaire was calculated as 1.05 (95% CI 1.05 – 1.22) using Youden’s J.

Data was analysed using the Statistical Package for Social Sciences (SPSS) version 16. The crude prevalence rate was determined by the total number of ET cases divided by the total population screened (3000) and reported as number of ET pr 1000 population. The age adjusted prevalence rate of ET in this study was determined using the World Health Organization (WHO) new world population standard [22].

## Results

### Demographic profile of the study population

**Figure 2:**
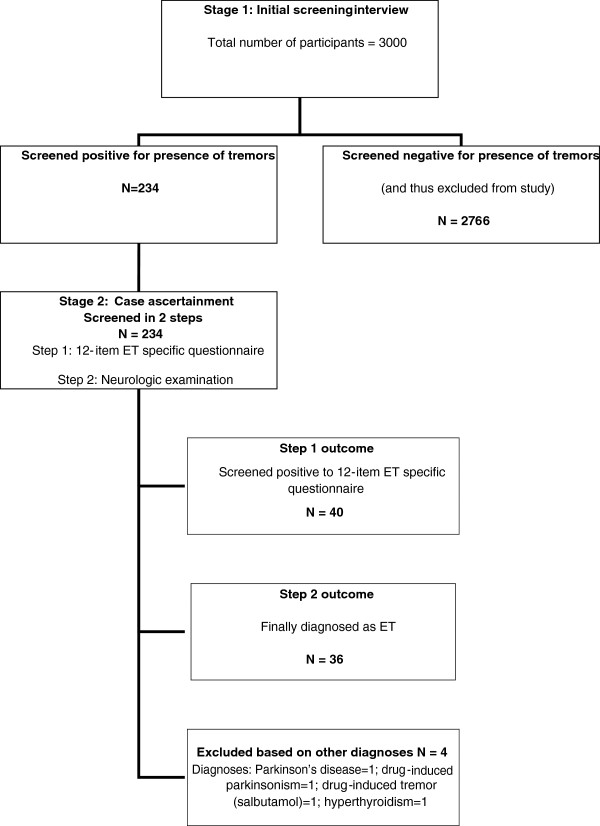
** Flow chart of participantevaluation and case ascertainment.**

A total of 3000 persons were screened for ET, comprised of 55.6% male (1668/3000) and 44.4% female (1332/3000). Age (by mid-decade strata) and gender distribution of the population screened is shown in Table 1. Figure 2 is a flow chart outlining further details of participant evaluation and case ascertainment.

**Table 1:** **Population distribution, age-specific andage–adjusted prevalence of essentialtremor per 1000 population**

**Age stratification**	**Population (%)**	**Gender**		**Number of ET cases**	**Age specific prevalence per1000**	**Age adjusted prevalence per1000 (WHO New Worldpopulation)**
		**Male (%)**	**Female (%)**			
**Below 15 years**	706 (23.5)	389 (13.0)	317 (10.5)	0	0	0
**15 to 24 years**	832 (27.7)	452 (15.1)	380 (12.6)	4	4.81	0.81
**25 to 34 years**	594 (19.8)	333 (11.1)	261 (8.7)	3	5.05	0.71
**35 to 44 years**	411 (13.7)	228 (7.6)	183 (6.1)	3	7.30	0.88
**45 to 54 years**	278 (9.3)	161 (5.4)	117 (3.9)	2	7.19	0.79
**55 to 64 years**	110 (3.7)	63 (2.1)	47 (1.6)	6	54.54	4.37
**65 to 74 years**	43 (1.4)	25 (0.8)	18 (0.6)	6	139.53	6.97
**75 to 84 years**	19 (0.6)	13 (0.4)	6 (0.2)	9	473.68	7.11
**85 years and older**	7 (.2)	4 (0.1)	3 (0.1)	3	428.57	2.14
**All ages**	**3000**	**1668 (55.6)**	**1332 (44.4)**	**36**	**12**	**23.78**

### Prevalence rates (crude and age adjusted) of essential tremor

**Figure 3:**
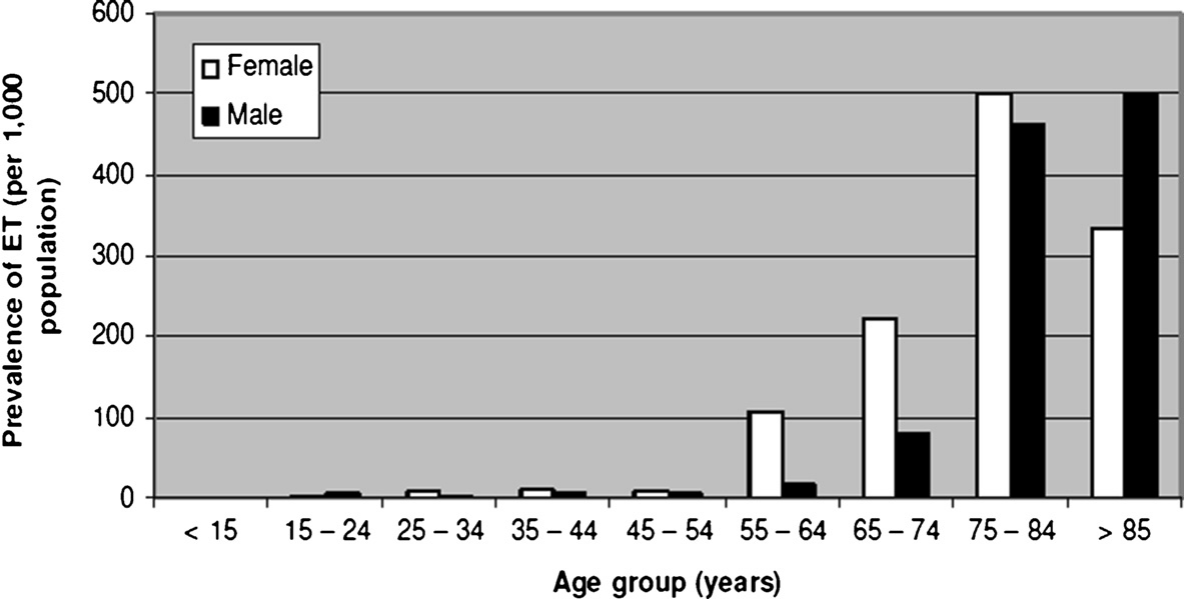
** Prevalence of essential tremorper 1,000 population byage strata and gender.**

A total of 36 of the 3000 screened participants were diagnosed as having ET, resulting in a crude prevalence rate of 12/1000 population (95% Confidence Interval – CI) = 8.1 – 15.9) (Table 1). The crude prevalence rates by gender were 10.2/1000 (i.e. 17/1668) in males and 14.3/1000 (19/1332) in females. The gender difference in crude prevalence was not statistically significant (relative risk 0.71, 95% CI 0.37 – 1.37, P = 0.40). Prevalence increased with advancing age from no cases documented in those below 15 years (0) and the highest rates in those between 75 and 84 years (473.7/1000). Figure 3 shows this increase in the prevalence of ET with advancing age in both sexes. ET prevalence was age standardized using the World Health Organization (WHO) New World Population standards as shown in Table 1 with a resultant age-adjusted prevalence of 23.8/1000.

### Demographic and clinical characteristics of persons with essential tremor

The mean age at study of ET cases was 59.6 ± 22.6 years while the mean age at onset of tremors was 44.6 ± 18.5 years. The male to female ratio was 1:1.1. The mean duration of ET was 4.9 ± 3.8 years while there was a positive family history of ET in 13 (36.1%) of the 36 ET cases. The most common initial symptom of ET was hand tremor in the 36 (100%) ET cases.

In 44.4% (16 of 36) the site of onset was the right arm, while 22.2% (8 of 36) started in the left arm and 10 of 36 (27.8%) had tremor onset in both upper limbs. The remaining 2 cases (5.6%) could not recollect the site of onset of ET. All 36 ET (100%) had bilateral tremors at the time of study, while none (0%) had head, voice or lower limb tremors.

All ET (100%) had postural tremors while 32 of 36 (88.9%) had kinetic tremors in addition to postural tremors. Only 1 out of 36 (2.8%) with ET had an intention tremor in addition to the postural tremor (with no other features of cerebellar disease). None of the ET cases had a rest tremor. Tremor severity was mild in the majority of cases (28 i.e. 77.8%), moderate in 7 (19.4%) and severe in 1(2.8%). Only 1 (2.8%) of the ET cases was on treatment at the time of this study (and the only one with a prior diagnosis of ET). 35 of the cases were newly diagnosed as a result of the study.

## Discussion

This study utilized a robust methodology to determine the prevalence of essential tremor in an urban black African community in sub-Saharan Africa, where data are sparse regarding the epidemiology of movement disorders including ET. The sample size was modest, and was dictated by financial and logistic constraints, as well as a commitment to allow complete case ascertainment using a manageable population size. The study instrument for screening for ET had several advantages, in that the sensitivity (100%), specificity (95.2%) and number needed to diagnose ET (1.05) significantly improved the likelihood of complete ascertainment of cases of ET in the participants. Furthermore, face-to-face neurological diagnosis remains the clinical gold standard for the diagnosis of ET, and was employed in this study to ensure diagnostic certainty.

The crude and age-standardized prevalence data obtained here fall within the range previously reported from other populations (4.0 – 39.2/1000), but are higher than the values from earlier studies based in a rural community in the same ethno-geographical zone, but published three decades ago by Osuntokun *et al.*[15]. The earlier study reported a crude prevalence rate (0.1/1000) ten times lower than the present study. Although there is a paucity of comparative data from other African countries, the studies by Haimanot *et al.*[16] (1990) and Dotchin *et al.*[17] (2008) reported lower prevalence rates of 5/100,000 and 82/100,000 respectively from communities in Ethiopia and Tanzania (both in east Africa) respectively. In contrast to these African studies, our prevalence rates are lower than that reported from a recent study in Sile, Turkey where the crude prevalence of ET was documented as 30/1000 in persons above 18 years of age [23]. It is also lower than the prevalence rate documented from a community based study in Manhattan New York [24].

However, direct comparison of these data presupposes that the population structures of these countries are similar, and the lack of age-adjustment in the earlier West African study [25] and that of Haimanot [16] limit the conclusions that can be derived regarding differences in prevalence. Though the study by Dotchin *et al.*[17] employed age – adjustment this was done to United Kingdom population of 2001 which may not reflect WHO new world population.

World-wide, the prevalence of ET varies from not being present at all [25] to being common [26]. Differences in prevalence estimates have been attributed to differences in study design, study population and methodology and diagnostic criteria. Many of the initial studies on ET prevalence relied on screening instruments only though in the last two decades most of the population studies have incorporated clinical examination.

Other reasons for varied worldwide prevalence could be differences in exposure to risk and aetiological factors. Aging is the most consistent documented risk factor associated with increasing prevalence of ET [27] a finding also corroborated by this study. Genetics have also been implicated in ET as demonstrated by clustering of the condition within families [28]. About a third of the participants with ET in this study had a positive family history of tremor in a first degree relative. It is conceivable that undiagnosed ET and recall bias would have lowered the reporting of a positive family history. Environmental factors (such as harmane) have been linked to ET, although the etiologic association is confounded by the possibility of a genetic risk modifier [29].

A gender predilection has previously been reported in some ET studies, while others such as ours have not found any male to female differences in the prevalence of ET [4,17]. The basis of a gender predisposition is unproven and there are no strong biological indications that could explain such findings. However, environmental peculiarities relating to occupational and recreational exposures differ by gender, and are possible contributors to gender differences.

The clinical phenotype of ET in this study is consistent with descriptions from other population-based studies. The predominant tremor location in our cohort is similar to previously documented reports with upper limb tremor being the most common feature. However, compared to other population based studies, none of our cohort had a head tremor. Dotchin *et al.*[17] in Tanzania found head tremor in 54% of their cohort, while Louis *et al.*[4] reported head tremor in 27% of their patients. There are some possible explanations for this discrepancy. Most importantly, the initial screening questions did not specifically ask for the presence of head tremor, but rather hand tremor, and so it is entirely feasible that this inadvertently screened out persons with isolated head tremor as the ET phenotype. However the second screening questionnaire specifically screened for head tremor in addition to other tremor locations. This was in addition to using a face to face examination to ascertain the nature of the tremor. In this case ascertainment stage, none of the 36 cases had combined head and hand tremors, all had hand tremors only. ET can manifest as isolated hand tremors, isolated head tremors, or a combination of hand tremors with head tremors. The number of ET cases encountered was small (36), and mainly of a mild nature (in about three quarters of the cases) and this could also partly account for the absence of the concomitant hand and head tremors. In general, in our clinical practice, we more commonly encounter isolated hand tremors, combined hand and head tremors, and less commonly isolated head tremors, and so our finding may simply be a reflection of the relative frequencies of these phenotypes. The higher proportion of mild cases in this study may indicate the greater likelihood of finding milder, less disabling ET in the community (in contrast to a clinic-based study).

We could not assess drug responsiveness in this study because of the low treatment rate (1/36 only on treatment) and a concomitant large treatment gap in our cohort with ET. As many as 75 - 99% of patients with ET detected through population-based studies are reported to be previously undiagnosed and untreated [4,30]. Our findings corroborate the previous documentation of a large treatment gap in ET in the developing world alluded to in a previous hospital-based study in a tertiary hospital in Nigeria which highlighted the low hospital frequency of ET over a 25-year period (only 10 ET cases out of 2.1million patients) and proposed a probable high treatment gap considering the presumption that ET is a relatively common disorder [31]. Several factors including differing severity and impact of ET on activities of daily living, non-recognition or poor awareness of ET as a neurological or medical disorder amenable to treatment, and limited access to care may contribute to this gap.

We found that a large proportion of ET cases reported that their tremors began on one side but however did not find any remarkable gross asymmetry in the tremor severity in any of the cases on clinical examination, We suggest that, as often encountered in the clinic setting, tremors may be more noticeable to patients in the dominant hand despite being present in both hands, because of the associated interference of tremors with carrying out actions, and thus attribute this finding to misperception of the nature of onset. Furthermore, due to the long standing nature of ET tremors, historical accounts of site of onset may not be accurate. Also, although ET is predominantly regarded as a symmetrical disease, there is clear evidence that it also presents asymmetrically, and indeed that mild asymmetry is a fundamental property of ET [32]. Putzke *et al.*, in a long-term follow-up study, reported that about 55% of ET cases in their series presented with asymmetrical disease, and that the occurrence of asymmetric ET may predict disease progression [33].

Our study has certain limitations relating to the size of the population screened and invariably, the population structure. The population screened (3000) is lower than that from previous studies, but we anticipate that the rigorous methodology employed ensured that our data are credible and truly representative of the current prevalence of ET in our community. We would have ideally screened a larger population, but were limited by the attendant cost implications. The proportions of persons in the age groups is a reflection of the Nigerian population structure which, according to current official statistics (2011 estimates), is comprised as follows (combined rural and urban figures): <15 years (40.9%), 15 – 64 years (55.9%), 65 years and above (3.1%), and median age (19.2 years) [34]. Our study was conducted in an urban centre and the smaller percentage of urban-dwelling elderly (>65 years – 2.2%) is expected. We however provide age adjustment to the WHO New World population and complete data for adjustment to any population structure to enable comparison of our findings with those of other researchers. Finally, we note the possibility of persons with very mild tremors having screened negative in the initial stage if the tremors were not obvious to the participants themselves.

## Conclusions

We conclude that ET is a common disorder in the urban Nigerian population studied here, and the clinical phenotype is similar to that previously described by other researchers. Improved public awareness and physician education will ensure that ET is recognized, and treatment offered where appropriate.

## Abbreviations

EAs: Enumeration areas; EPISIL: Epidemiology of stroke in Lagos; ET: Essential tremor; LGA: Local government area; MDS: Movement disorders society; NND: Number needed to diagnose; SPSS: Statistical package for social sciences; WHO: World health organization.

## Competing interests

The authors declare no competing financial or non-financial interests.

## Authors’ contributions

NUO was responsible for the study concept and design, data analysis and manuscript editing. IAB contributed to the study design, data analysis and manuscript preparation. OOO contributed to manuscript preparation and manuscript editing. FIO and MAD contributed to conceptualization, manuscript editing and review. The final copy has been read and approved by all contributing authors. All authors read and approved the final manuscript.

## Pre-publication history

The pre-publication history for this paper can be accessed here:

http://www.biomedcentral.com/1471-2377/12/110/prepub
